# Adiponectin: A player in the pathogenesis of hormone-dependent cancers

**DOI:** 10.3389/fendo.2022.1018515

**Published:** 2022-10-06

**Authors:** Alexandra Tsankof, Konstantinos Tziomalos

**Affiliations:** First Propedeutic Department of Internal Medicine, Medical School, Aristotle University of Thessaloniki, AHEPA Hospital, Thessaloniki, Greece

**Keywords:** adiponectin, adiponectin receptors, obesity, breast cancer, cervical cancer, endometrial cancer, ovarian cancer, prostate cancer

## Abstract

Hormone-dependent cancers are a major cause of morbidity and mortality in both genders. Accumulating evidence suggest that adiponectin, an adipokine with multifaceted functions, is implicated in the pathogenesis of several malignancies. In the present review, we discuss the existing data regarding this relationship. Several observational studies showed that low adiponectin levels are associated with higher risk for breast, cervical, endometrial, ovarian and prostate cancer. A relationship between adiponectin and the aggressiveness of some of these tumors has also been reported. *In vitro* studies reported that adiponectin inhibits the proliferation and induces apoptosis of breast, cervical, endometrial, ovarian and prostate cancer cells. Given the high prevalence of these cancers and the substantial associated morbidity and mortality, the role of agents that increase adiponectin levels and/or stimulate its activity should be evaluated for the prevention and management of these common tumors.

## Introduction

**Figure 1:**
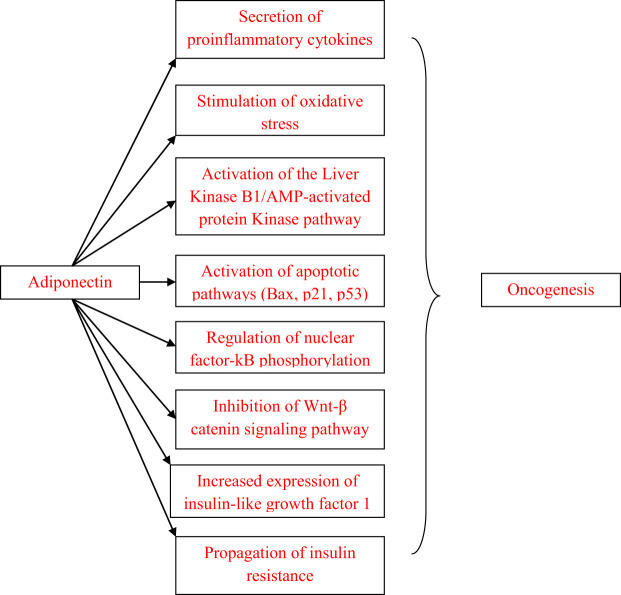
Pathways linking adiponectin and oncogenesis.

Obesity is an established risk factor for several malignancies, including colon, breast, endometrial, prostate and ovarian cancer (1–3). Metabolic abnormalities, particularly insulin resistance, as well as chronic subclinical inflammation are frequently present in obese patients and can induce adipose tissue dysfunction, which in turn appears to play a role in obesity-related oncogenesis (4). Adipose tissue is the largest endocrine system and actively participates in the regulation of metabolism, inflammation, fat distribution, appetite and bone structure (4). Adiponectin, a protein hormone secreted by adipocytes, participates in glucose regulation but also exerts anti-inflammatory, antiatherogenic and cardioprotective effects as well as effects on lipid metabolism and fatty acid oxidation (5). Accumulating preclinical and clinical data suggest that adiponectin contributes to the pathogenesis of a wide variety of malignancies, especially hormone-dependent cancers (**Figure 1**). This review aims to summarize the links between adiponectin and the most frequent hormone-related malignancies, namely breast, cervical, ovarian, endometrial and prostate cancer.

## Adiponectin: Physiology and oncogenesis-related functions

Adiponectin is predominantly secreted by adipocytes in white adipose tissue but is also secreted in lesser amounts by other tissues such as brown adipose tissue, colon, ovaries, salivary glands, liver and skeletal muscle suggesting an autocrine and/or paracrine function upon other tissues. It is a 244-amino acid protein that includes an amino-terminal signal peptide, a species-specific variable domain, a collagen-like region of 22 Gly-X-Y repeats and a carboxyl-terminal globular domain that binds to adiponectin receptors, and is similar to the C1q (Complement factor 1q) and the trimeric topology of tumor necrosis factor α(TNF-α) (5).

The adiponectin collagen-like region allows oligomerization of the protein as a trimer though disulfide bonds and hydroxylation and glycosylation of four conserved lysine residues, while this enables the formation of middle molecular weight (MMW) examers and high molecular weight (HMW) multimers. Circulating adiponectin can take the form of full-length or HMW adiponectin in plasma, LMW and gAd (globular adiponectin), which are also present in low concentrations due to their shorter half-life (6).

Adiponectin levels are modulated by a variety of environmental, physiological and pharmacological factors such as nutrition, hormones, inflammation and medications (6). Testosterone, growth hormone (GH), glucocorticoids and prolactin decrease adiponectin secretion (7).

Three adiponectin receptors have been identified, namely adiponectin receptors 1 and 2 (AdipoR1 and AdipoR2) and T-cadherin. AdipoR1 and AdipoR2 are 375- and 311-amino acid proteins, respectively, with a molecular mass of 42.4 and 35.4 kDA, respectively. They are structurally highly related, as their protein sequence shares 67% identity, and include7 transmembrane domains, an extracellular carboxy-terminus and an intracellular amino-terminus, a structure that is opposite to that of all other G-coupled protein receptors. AdipoR1 and AdipoR2 are also highly conserved, since they share 95 and 97% identity between mice proteins, respectively (8–10).

Adiponectin exerts insulin-sensitizing effects in both skeletal muscle and in adipose tissue by enhancing APPL-1/AMPK interaction, which in turn increases the glucose uptake through glucose transporter 4 (GLUT4) (5). Therefore, low levels of adiponectin might increase the risk for oncogenesis, given that insulin resistance is associated with higher risk for several malignancies (4). Adiponectin inhibits fibroblast growth factor-2 (FGF-2)-stimulated endothelial cell proliferation and decreases vascular endothelial growth factor (VEGF)-induced endothelial cell migration. Intralesional injection of recombinant murine adiponectin into hypervascularized murine fibrocarcinomas led to a 60% reduction in tumor volumes and weights accompanied by an increase in tumor apoptosis mediated through caspase-3 activation, which suggests that adiponectin could act as a potent angiogenesis inhibitor that can activate apoptosis, and thus inhibit tumor growth (11). Adiponectin exerts its effects mainly by the Liver Kinase B1/AMP-activated protein Kinase (LKB1/AMPK) pathway, which inhibits signaling pathways involved in cell cycle initiation, cell growth and survival such as extracellular signal-regulated kinases 1/2 (ERK1/2), phosphatidyl-inositol 3-kinases (PI3K)/Protein Kinase B (Akt), c-Jun N-terminal kinase (cJNK) and signal transducer and activator of transcription-3 (STAT3) (12). Adiponectin also regulates the expression of different proteins involved in cell cycle and apoptosis, including up-regulation of p53 and Bax and down-regulation of c-myc, cyclin D1 and Bcl-2 (13). Moreover, adiponectin has anti-inflammatory effects by suppressing the phosphorylation of nuclear factor kB (NF-kB), a transcription factor involved in the regulation of the activity of various pro-inflammatory cytokines (14).

## Adiponectin and breast cancer

Breast cancer is the most frequent cancer in females and accounts for 11.7% of all new cancer cases diagnosed in European countries and for 28.7% of all new cancers diagnosed in women. Among women, breast cancer accounts for 1 in 4 cancer cases and for 1 in 6 cancer deaths, ranking first in incidence in the vast majority of countries (15). Obesity is associated with increased incidence of breast cancer and with increased risk for more aggressive breast cancer (16). Regarding the relationship between adiponectin and breast cancer, lower circulating total and HMW adiponectin levels are associated with a higher risk of breast cancer, independently of body mass index (BMI) (17, 18). Recent data suggest that low adiponectin levels are also related to shorter overall mortality in patients with breast cancer (19). Moreover, in premenopausal women, lower plasma adiponectin levels predict the progression from intraepithelial neoplasia to invasive breast cancer independently of age and BMI (20). Adiponectin receptors have been identified in breast cancer cells (18). In T47D breast cancer cell lines, adiponectin inhibited cell proliferation and reduced viability, an effect partially mediated by the activation of ERK1/2 (18). In MDA-MB-231 breast cancer cells, adiponectin arrested cell cycle progression and induced apoptosis by suppressing the glycogen synthase kinase 3β/β-catenin signaling pathway (21). Adiponectin also inhibited the proliferation of MCF-7 breast cancer cells by reducing the translation of genes involved in cell cycle regulation (mitogen-activated protein kinase 3 and ATM) and apoptosis (BAG1, BAG3 and TP53) (22).

## Adiponectin and cervical cancer

Cervical cancer is the fourth most frequently diagnosed cancer and the fourth leading cause of cancer-related death in women, with an estimated 604,000 new cases and 342,000 deaths worldwide in 2020 (15). Obesity is an established risk factor for cervical cancer and is also associated with more aggressive forms of this malignancy (23). There are very limited data regarding the role of adiponectin in the pathogenesis of cervical cancer. An *in vitro* study showed that adiponectin receptors are expressed in cervical cancer HeLa cells and that their expression increases in adiponectin-treated cells (24). Moreover, adiponectin inhibited the proliferation of HeLa cells, as evidenced by a significant increase in the cell population in G0/G1 phase, concomitant with a reduction of cell number in S and G2/M phases (24). A down-regulation of cell cycle regulators, namely cyclin D1 and c-myc, was also observed, along with an activation of apoptosis, mediated by the enhanced expression of p21, p53 and Bax and reduced expression of Bcl-2 (24).

## Adiponectin and ovarian cancer

Ovarian cancer is the 8th most commonly occurring cancer in women and the 18^th^ most common cancer overall. There were more than 313,000 new cases of ovarian cancer in 2020 (15). The relationship between obesity and ovarian tumor development has become increasingly evident, particularly in post-menopausal women (25). In case-control studies, women with low levels of adiponectin have higher risk for ovarian cancer (26, 27). Adiponectin receptors are expressed in both epithelial and granulose ovarian cancer cells (28) and their expression is associated with more advanced cancer and shorter progression-free and overall survival (29). Moreover, adiponectin inhibits the growth of OVCAR-3 and SKOV-3 ovarian cancer cells and antagonizes the proliferative effects of 17β-estradiol and insulin-like growth factor-1 on these cells by down-regulating the expression of their receptors (28). Stimulation of adiponectin receptors was also shown to induce G1 cell cycle arrest and promote apoptosis in OVCAR3, OVCAR4 and A2780 ovarian cancer cells (30).

## Adiponectin and endometrial cancer

Endometrial cancer is the sixth most commonly diagnosed cancer in women with 417,000 new cases and 97,000 deaths recorded in 2020 (15). Low circulating adiponectin levels are associated with higher endometrial cancer risk, independent of other obesity-related risk factors, and this relationship appears to be stronger in premenopausal women (31, 32). Adiponectin receptors are expressed in both normal human endometrium and in endometrial cancer tissues (33). Adiponectin was shown to decrease the proliferation of KLE and RL95-2 endometrial cancer cells and also reduces their adhesion and migration by activating the adaptor molecule LKB1 (33). In HEC-1-A endometrial cancer cells, adiponectin inhibited cell growth and induced apoptosis by inactivating Akt and decreasing cyclin D1 expression (34). More recently, adiponectin was shown to promote the development of endometrial cancer by activating mitogen-activated protein kinase (MAPK) (35).

## Adiponectin and prostate cancer

Epidemiological data suggest that low adiponectin levels might also be associated with higher incidence of prostate cancer (36, 37). Single nucleotide polymorphisms of genes that encode adiponectin and AdipoR1 are also related to prostate cancer incidence and aggressiveness (38, 39). Adiponectin receptor expression is lower in prostate cancer tissue than in benign prostate tissue (39, 40). *In vitro* studies also showed that adiponectin inhibits the proliferation of PC-3 prostate cancer cells (41) and also suppresses angiogenesis by inhibiting m-TOR activation of VEGF (42). Adiponectin also suppresses oxidative stress in human 22Rv1 and DU-145 PC cell lines by increasing the expression of NADPH oxidase-2 and -4 (43).

## Conclusions

Both *in vitro* and observational studies suggest that adiponectin is potentially implicated in the pathogenesis of several hormone-related malignancies. Given the high prevalence of these cancers and the substantial associated morbidity and mortality, the role of agents that increase adiponectin levels and/or stimulate its activity should be evaluated for the prevention and management of these common tumors.

## Author contributions

AT drafted the manuscript. KT edited and critically revised the draft. All authors contributed to the article and approved the submitted version.

## Conflict of interest

The authors declare that the research was conducted in the absence of any commercial or financial relationships that could be construed as a potential conflict of interest.

## Publisher’s note

All claims expressed in this article are solely those of the authors and do not necessarily represent those of their affiliated organizations, or those of the publisher, the editors and the reviewers. Any product that may be evaluated in this article, or claim that may be made by its manufacturer, is not guaranteed or endorsed by the publisher.
